# Dental Caries

**Published:** 1883-05

**Authors:** W. D. Miller

**Affiliations:** Berlin


### DENTAL CARIES.

BY DR. W. D. MILLER, OF BERLIN.

I have for some time been impressed with the idea that we are all, so to say, afloat in our investigations upon the subject of caries, and my object in this article is to make a survey of the situation, and to find out how the matter stands at present.

The principal feature in all the publications which have as yet been made on this subject seems to me to be a lack of method, and perhaps in some cases an improper conception of the question to be solved.

Many observers have been hunting after "Bacteria," upon whom we could saddle all the blame, without any further examination into the innocence or guilt of these organisms. I have hunted them day and night, and have found enough of them, but I have come to the conclusion that a satisfactory explanation of the cause of caries can never be obtained through the microscope alone.

In general, in conducting any investigation of the lower forms of life, with a view to determining their power of producing disease, the following points must be considered:

1st. Are the organisms in question at all pathogenic—i. e., can they produce disease?

2d. Can they be transmitted from one person to another, or, in other words, is the disease produced by them an infectious one?

3d. In what manner do they find their way into the animal body?

4th. What are the physiological characteristics and history of the organisms, and how are they affected by substances which impede or prevent the development of lower organisms?

Up to the present time, however, little has been done towards solving any of these questions; the organisms found in and upon the teeth have been thrown into a lump and called Bacteria, or Germs, and the fact of their being in the dental tubuli has been considered sufficient reason for looking on them as pathogenic and infectious, and as producing all the phenomena of dental caries.

We find within the human mouth—

1st. Leptothrix buccalis,
2d. Vibrio regula,
3d. Clostrydium butyricum,
4th. Mycoderma aceti,
5th. Saccharomyces mycoderma,
6th. Spirochete dentium (not plicatilis), not to speak of the Leptothrix pusilla, Penicillium microsporum, etc., of Klebs (the former of which he considers to be concerned in the production of tartar), or of the Bacterium subtile, Bacterium termo, and various other forms of vegetable life, which undoubtedly may be occasionally met with in the human mouth. Now do all these keep gnawing at the teeth, or is there any one of them which is to be looked upon as particularly destructive? In earlier writings upon this subject, attention was paid only to Leptothrix buccalis, or rather to the leptothrix form of this fungus, while of later writers some speak of a certain bacterium of the form of a half U, others of micrococci, and yet others are content with the simple appelation of germs.

It seems to me to be of the greatest importance to determine; 1st, what species of fungus we have to deal with; and 2nd, the morphology, and physiological characteristics of this fungus; for, until we have done this, a satisfactory solution of the problem of caries must remain an impossibility.

I look upon the Leptothrix buccalis as the chief agent in the production of caries. I hope at another time to enter fully into the morphology, etc., of this fungus, so I will now only state that it produces not alone threads, but bacilli, bacteria, micrococci, and most likely screw forms, and that it is the coccus form which is most destructive to tooth tissue. As for Vibriones, they do not appear to perform any part until the decay is in the very last stages, and may practically be neglected altogether.

Respecting the numbers and frequency with which the 3rd and 4th named species occur in the human mouth, very little is known, it being almost impossible, except through certain characteristic involution forms which they build, or by means of the culture method, to distinguish them from Leptothrix buccalis. Here there seems to be room for investigation. Both of these fungi create an acid reaction of their substratum, the first producing butyric, the second acetic acid; should they, therefore, develope to any considerable extent in any part of the oral cavity, they would unquestionably prove injurious to the teeth in that part. It is, however, doubtful whether these fungi have more than a transitory existence in the human mouth. The existence of Saccharomyces mycoderma I demonstrated some months ago. Since then I have found this fungus in many more preparations; very often the cells become so elongated and thin that they are easily overlooked and set down for leptothrix threads.

I think there can be no question of the evil designs of this organism. Of course it is not so mischievous as Leptothrix buccalis, but we are reminded that we must keep our eyes open, for there may be some things about caries of which we have not yet dreamed. It is hardly necessary to speak of the other species of fungi which I have enumerated, so I will return to the Leptothrix buccalis.

The question of the pathogenic character of this fungus has, I think, been clearly settled. Numerous cases are on record which show, that starting out from the mouth it may penetrate the most various organs of the human body, and everywhere be as destructive as it is to the teeth.

But what role does it play upon the teeth ? It is here that our lack of knowledge of the real nature of the fungus makes itself so severely felt. If we were able to say with certainty that it generates an acid or an alkali, or that it leaves its substratum neutral, we would be spared a great deal of unnecessary work and thinking. I have made a great number of experiments with a view of determining this question, and the results have been of a negative character, for, as I have not yet succeeded in producing a pure culture of this fungus, I attach very little value to these results If I am asked whether I think that acids are instrumental in producing caries of the human teeth, my answer is decidedly affirmative. I would not say in *all* cases, but in a very great many. The fact that a mixture of starch-containing food with saliva, at the temperature of the human mouth, generates in a space of four to six hours acids sufficiently strong to soften tooth tissue, is absolutely undisputable. The fact that exactly these conditions are continually to be found in the human mouth is also unquestionable. It follows, therefore, most clearly, that a softening of the tissue of the tooth must take place in the human mouth, when the conditions specified above are present. This softening produced by a withdrawal of lime salts, or by a rupture of the bond of union between the organic and inorganic portions of the tooth, at once lays it bare to the attack of micro-organisms.

The question of acids, however, I would like to discuss at another time. I wish now to consider only the work of the organisms (Leptothrix buc.) as revealed by a microscopic study of at least a score of hundreds of microscopic sections of carious dentine, subjected to at least a dozen different modes of staining. I have made use of five different methods in procuring my preparations:

*a.* From a freshly extracted tooth containing a large amount of carious dentine, as large a piece as possible is removed by means of a spoon-shaped excavator; from this, microtome sections are made, some parallel with, others at right angles to the dental tubules.

*b.* Sections are sawed from carious teeth comprising both the carious and sound part; these, after hardening in alcohol, may be ground sufficiently thin for both the normal and carious parts to be examined with high powers of the microscope.

*c.* From a carious tooth (freshly extracted, of course,) all the softened dentine is removed *with the greatest care* and ground sections prepared.

*d.* Ground sections are made from dentine which has been kept at the temperature of the human body, in septic liquids, and in contact with carious dentine since May, 1882.

*e.* Sections are made from apparently sound, freshly extracted teeth.

The sections are treated in each case for a short time with absolute alcohol, and afterwards stained and mounted in Canada balsam, or in a concentrated solution of acetate of potassium; (glycerine may not be used, as in case of some analine dyes at least, it abstracts the coloring matter.) I have used Magdala, Methylene-blue, Phenylene-blue, Fuchsin, Methyl-violet, Bismarck-brown, etc., etc., etc.

Sections prepared as indicated under (*a*), appear under the microscope as described in the "Dental Cosmos" for January of this year. Such specimens should always be first examined under a very low power, say 40 diameters, as in this way the distribution of the organisms in the section may be seen at a glance. If the piece of dentine from which the section was made comprised anything like nearly all the softened dentine in the cavity, we will find in the section, almost always, large tracts which contain no organisms, and in which the dentine is perfectly regular and shows no anatomical changes whatever.

In sections cutting the tubules at right angles, I frequently find fields containing from 8,000 to 10,000 tubules, of which not more than half a dozen contain micro-organisms; in other large fields the micro-organisms are strictly confined to the tubules.

The question suggested by these cases is this: what produced the softening of dentine in those parts which are free from micro-organisms? I, for my part, know of no way in which this softening can be accomplished, except by acids. I do not say that there may not be other agents, neutral or alkaline, which can accomplish this; I only say, we know of no other.

Whether the micro-organisms themselves assist in generating the acid which produces the softening of the dentine, can, as I have before remarked, be determined only by the pure culture.

Sections prepared in the manner described under (b), very frequently unmistakably show a zone of softened, not infected, dentine, intervening between the normal and infected dentine, nor have I as yet found a case where I could be sure that the organisms crossed from the softened into the perfectly sound tissue; most certainly not sufficiently so to produce any anatomical changes. This is a point which requires very careful study, as it is sometimes almost, if not altogether, impossible to determine the boundary between the carious and normal dentine, and even those cases in which they appear to have passed into the normal dentine, or rather into the tubules of the normal dentine, may be easily misinterpreted. The diameter of the dental tubules is considerably greater than that of most micro-organisms found in the oral cavity. When, therefore, the open ends of the tubuli are exposed, a coccus might enter it just as he might enter a glass tube of the same diameter. In the former case, no more than in the latter, does the mere presence of the organisms give us the slightest right to attribute to them the power to destroy either the one tube or the other.

This is a fact which I think has been entirely disregarded. A bacillus found in the sputum of a certain person does not prove that person to be a sufferer from tuberculosis; a micrococcus found in a vein of the kidney does not establish a case of abscess; neither does a micro-organism of any sort found in a tubule of apparently sound dentine prove that that dentine is or ever will be carious; nor does the finding of micro-organisms in the tubules of softened, so-called carious dentine, entitle us to attribute the softening to their action.

Not until we see definite anatomical changes in the dentine itself, directly traceable to the action of the micro-organisms, is it allowable to look upon them as a factor in producing the caries. Beyond that all is, as yet, simple guesswork.

In many cases the discoloration of the dentine is a guide, but this does not always answer.

There are various kinds of bacteria which have the power of forming a coloring matter, soluble in the medium in which they vegetate, so that not only the organisms themselves as seen *en masse*, but their substratum as well, acquire a deep red, blue, violet, yellow, or other color. But in the thousands of sections of carious dentine which I have made, I have never yet met with anything in any way entitled to be called a pigment bacterium, nor am I aware that any observer who has made an exhaustive study of the different species of micro-organisms fonnd in the human mouth, has ascribed to them any power to produce pigment. Thin sections of very carious dentine show, even to the naked eye, the perfectly colorless patches of micro-organisms. Moreover, I have never seen any color in carious dentine which I have not repeatedly produced by the action of acids, or exposure to the air.

Specimens prepared as indicated under (*c*), contain very often, not a trace of an organism. It requires a great amount of care to be sure that you have removed all the softened dentine and no more, as a layer $\frac{1}{100}$ m. m thick, left in the bottom of the cavity, might be sufficient to destroy the value of the experiment. I use for this purpose a blunt spoon-shaped excavator.

Specimens prepared according to (*d*), show that a limited number of organisms have entered the tubules, a fact of little importance, because, as above suggested, if we should fill a system of glass tubes of the same diameter as the dentinal tubes with organic matter, and place it for some months in a septic liquid, a section made parallel to and through the middle of one of the tubes would most likely reveal micro-organisms of some kind, but we would scarcely accuse them of an attempt upon the glass itself.

I suppose a coccus could enter a dead dentinal tubule about as readily as a glass tube of the same diameter; to enter a living tubule he must, of course, first overcome the resistance offered by the dental fibril, which he undoubtedly can do. This does not signify that he can in any way attack the unchanged basis substance. As a matter of fact, the sections under consideration show no anatomical changes whatever; no expansion of the tubules, no breaking through of the basis substance, or any other structural change. Examined microscopically they do not appear to be changed in the least, the edges and corners being as sharp and hard as when they were put in the flasks nine months before, although I have taken care, by frequently adding fresh carious dentine, that the state of the liquid should be as septic as anything ever found in the human mouth, and much more so, and have kept them at an agreeable temperature (cold, hard dentine doesn't seem to be to their taste), yet they seem to have made no impression upon the pieces used.

Again, if we examine a freshly extracted carious tooth, we will find, say on the grinding surface, the enamel broken through and the dentine softened to the depth of 1 to 2 m. m. Now let us suppose that this whole process has been caused by fungi alone. If, then, we place the tooth under conditions of moisture and temperature favorable to the development of these fungi, the carious process should continue. The enamel should dissolve away more and more, and the dentine become softened deeper and deeper.

This experiment I have repeatedly tried, and have invariably found that after a lapse of from three to five weeks, the dentine which was previously softened has become entirely consumed. The softening has, however, entirely ceased, and the walls are found to be perfectly hard, and if the cavity is syringed out with water it will be found to be as clean as though some scrupulous dentist had prepared it for filling. In other words, we obtain a result entirely contrary to our expectations and as unlike ordinary caries as it could well be.

I have made but four microscopic preparations from such teeth. Three of them do not contain a single coccus which may not be called accidental, while the other shows half a dozen tubules containing as many cocci as might be counted on one's fingers, the tooth not having remained sufficiently long in the liquid for all the softened dentine to be consumed.

It appears to me, on the whole, that the idea that any Tom, Dick or Harry of the world of fungi, who happens to get into the human mouth, at once sets to work to pick the teeth to pieces, is without much show of reason, and to make caries absolutely dependent upon these same agents is still more questionable.

Preparations of the kind indicated under (*d*), are sometimes very instructive. I will describe only one case. A section was ground from a tooth which had been over thirty years in the human mouth. The section revealed a circle of interglobular spaces, extending completely around the pulp cavity just beneath the enamel. Through a deep, broad fissure in the enamel, cocci had gained access to the interglobular spaces, and, passing from one to the other, had filled every space for full three quarters of the distance around the section. Whether they had made that their home for one, ten or twenty years, it is not possible to say. At any rate the dentine did not appear to have suffered in the least from their presence, and only now and then a few cocci appeared to have forced an entrance into the dentinal tubuli for a fraction of a millimeter, without in any way encroaching upon or invading the basis substance.

I have said nothing as yet about enamel, which, whatever may be the cause of caries, is the first and strongest barrier to be overcome. I have a large number of preparations of carious teeth, comprising both the enamel and dentine, and I can only say that as far as I am at present able to judge, indications of a purely parasitical caries of the enamel are very meagre indeed.

To sum up my views of the etiology of *caries dentium* then, I believe as follows:

1st. Caries of the teeth is not entirely of a parasitic nature.

2d. The first stage of dental caries consists in a softening of the tissue of the tooth.

3d. In effecting the softening, acids, particularly those generated by fermentation, often play a very important part.

4th. Whether micro-organisms are or are not concerned in the production of the first stage of caries, or whether any other agent is concerned in the process, (be it in the form of a simple solvent, or be it a pathological action on the part of the tooth itself,) are questions which certainly, as yet, cannot be satisfactorily answered.

5th. Destruction of the enamel may take place quite independently of micro-organisms.

6th. The second stage of caries, devitalization and breaking down of the softened tissue, is entirely parasitic, and is inaugurated chiefly, if not wholly, by the elements of the Leptothrix buccalis, while later on various other fungi may take part in the decomposing process.

From this it may be seen that I am a believer in acids, a believer in micro-organisms, and a believer in an unknown cause; that is, I believe that there are agents active in the production of caries which are yet to be discovered.

I have attempted in this hurriedly-written article, not so much to point out what the fungi of caries can do, as what they do not do, or rather what my preparations do not prove that they do, for although their powers for evil are very great, yet I believe that there is a tendency to overestimate them, and to jump to conclusions which can hardly be said to be sufficiently well supported by facts.

